# Long-term radiographic assessment of maxillary sinus floor augmentation using beta-tricalcium phosphate: analysis by cone-beam computed tomography

**DOI:** 10.1186/s40729-016-0042-6

**Published:** 2016-04-01

**Authors:** Tsuneji Okada, Toru Kanai, Noriko Tachikawa, Motohiro Munakata, Shohei Kasugai

**Affiliations:** 1 Clinic for Implant Dentistry, Dental Hospital, Tokyo Medical and Dental University, 1-5-45 Yushima, Bunkyo-ku, Tokyo, 113-8549 Japan; 2 Oral Implantology Department of Prosthodontic Dentistry for Function of TMJ and Occlusion, Kanagawa Dental University, 82, Inaokachou, Yokosuka-shi, 238-8580 Kanagawa, Japan; 3 Oral Implantology and Regenerative Dental Medicine, Department of Masticatory Function Rehabilitation, Division of Oral Health Sciences, Graduate School of Medical Dental Sciences, Tokyo Medical and Dental University, 1-5-45 Yushima, Bunkyo-ku, Tokyo, 113-8510 Japan

**Keywords:** Beta-tricalcium phosphate, Maxillary sinus floor augmentation, CBCT analysis

## Abstract

**Background:**

The long-term stability of maxillary sinus floor augmentation with β-TCP remains largely unknown. We report the long-term assessment of volumetric changes in maxillary sinus floor augmentation with β-TCP by cone-beam computed tomography (CBCT).

**Methods:**

The subjects included 30 patients who underwent maxillary sinus floor augmentation using β-TCP and 58 implant placement for unilateral maxillary defect, simultaneously. Volumetric changes in β-TCP and the height of peri-implant bone were analyzed by CBCT.

**Results:**

In all patients, the mean volume of the grafted bone decreased from immediately after implant placement to 6 months after implant placement (75.6 % reduction rate); it decreased further at 2.5 years after implant placement (54.9 % reduction rate). The mean of the height from the implant tip to the maxillary sinus floor was 2.00 ± 1.51 mm, 0.73 ± 1.33 mm, and −0.72 ± 1.11 mm immediately, 6 months, and 2.5 years after implant placement, respectively. The implant tip protruded beyond the maxillary sinus floor in approximately 70 % of the implants (41/58 implants) at 2.5 years after surgery. During the observation period, the implant survival rate was 100 %.

**Conclusions:**

The radiographic analysis by CBCT is considerably more advanced than previous radiographic examinations. Although maxillary sinus pneumatization continues to progress ≥1 year after maxillary sinus floor augmentation with β-TCP, it stabilizes 3 years after surgery.

## Background

The maxillary sinus gradually expands after birth and becomes fully pneumatized with the eruption of all permanent teeth. Although the physiological cause and maxillary sinus pneumatization are largely unknown, it is believed that genetics, atmospheric pressure, and hormones are involved in it. This sinus is closely related to the root apex of the premolar and molar teeth, and it is either separated from the teeth by a thin layer of bone or its mucous membrane is in direct contact with the teeth. The pneumatization of the maxillary sinus and the reduction in the bone wall thickness resulting from the loss of teeth is attributed to atrophy caused by a reduction in stimulation to the bone. It is also believed that physiological effects of the mucous membrane of the maxillary sinus as well as factors such as osteoclasts and loss of tooth root resistance to atmospheric pressure in the maxillary sinus are considered to play a role [1–3].

When an implant is placed in the maxillary molar region with atrophy, the maxillary sinus floor is close to the alveolar crest. Thus, some cases may require bone grafting for maxillary sinus floor augmentation. Initially, the gold standard for bone grafting material used to perform this procedure was autogenous bone. The advantages of autogenous bone graft are that it has osteogenic, osteoinductive, and osteoconductive properties. It has been reported that the release of growth factors, such as platelet-rich plasma and transforming growth factor-β and early vascularization from the donor bone enables remodeling within 4–6 months after implant placement. Although the transplanted bone used in maxillary sinus floor augmentation varies, it has been reported that approximately 1.5 cm^3^ is required to elevate the sinus floor by 10 mm. This requires donor sites with sufficient bone mass (e.g., the mental region and mandibular ramus in the oral cavity or the iliac crest and tibia outside the oral cavity), and it places great stress on both the practitioner and the patient [4, 5]. In recent years, maxillary sinus floor augmentation has been introduced using various bone grafting materials to decrease invasiveness. In Japan, xenogeneic bone grafts are not permitted for ethical reasons; instead, synthetic bone grafting agents that are not derived from animals, such as hydroxyapatite (HA) and β-TCP, have been used. However, unlike nonabsorbable materials such as HA, β-TCP has properties to get absorbed in the body and to be replaced by the bone; in addition, its usefulness has been reported in orthopedic surgery and maxillofacial surgery [6, 7]. We have achieved good outcomes using β-TCP as the bone grafting material for maxillary sinus floor augmentation. However, the predictability of the material and the change in absorption of the transplanted material mass remain largely unknown.

Radiographic examinations to determine changes in bone mass in maxillary sinus floor augmentation have been conducted primarily using panoramic radiography [8–12], which allows for the assessment of the height of the maxillary sinus in only two dimensions. In addition, it does not allow for detailed examination or measurement of the interior of the maxillary sinus without factoring in magnification or distortion. Hounsfield developed X-ray computed tomography in 1982. In 2001, cone-beam computed tomography (CBCT), the features of which included low exposure and high spatial resolution, became available in the clinical setting. Shortcomings of CBCT are that it delivers inferior spatial resolution of soft tissue compared with conventional CT and does not offer the quantification of CT values. However, narrowing the field of view (FOV) decreases the amount of radiation exposure. Moreover, its greatest advantages include isotropic spatial resolution (voxel) and high spatial resolution (superior resolution of bone), which allow for the accurate measurement of the interior of the maxillary sinus.

The purpose of this study was to examine the effect of bone grafting material resorption on volumetric change and the predictability of the long-term progress of sinus floor augmentation using β-TCP by performing radiographic examinations using CBCT.

## Methods

This study is a prospective observational study. The subjects included patients who had undergone maxillary sinus floor augmentation using β-TCP and implant placement at the Clinic for Implant Dentistry, Dental Hospital, Tokyo Medical and Dental University during the 3-year period from January 2009 to December 2011. All patients underwent maxillary sinus floor augmentation at the same time as implant placement.

### Patient selection

Patients were excluded from the study if they met the following criteria:

- They were completely edentulous and believed to be affected by postoperative denture wearing.
- They required sinus floor augmentation and buccal onlay graft at the same time for narrow bone width or if they required large-scaled bone graft or floor augmentation, such as that for guided bone regeneration and split crest.
- They had insufficient occlusal clearance or extremely poor tooth crown-to-root ratio, indicated by a Cawood and Howell classification V.
- They had a history of diabetes, osteoporosis, bisphosphonate administration, immunological diseases, such as rheumatoid arthritis, chronic sinusitis, or otolaryngological problems, as well as other untreated systemic disease, or were smokers.

The informed consent was obtained from all the patients regarding bone grafting material use, the surgical procedure, as well as the multiple CBCT scans. This study was carried out with the approval of the ethics committee of the School of Dentistry.

### Surgical procedure

Maxillary sinus floor augmentation was performed by a skilled surgeon using the technique according Boyne et al. [13]. Patients underwent preoperative examinations, after which maxillary sinus floor augmentation and implant placement were performed simultaneously. The grafting material used was OSferion (Olympus Terumo Biomaterials Corp., Japan) composed of β-TCP (Ca_3_PO_4_)_2_ (calcium phosphate). Second-stage surgery was performed 6 months after implant placement, and prosthetic treatment was performed.

### Radiographic examination

**Fig. 1:**
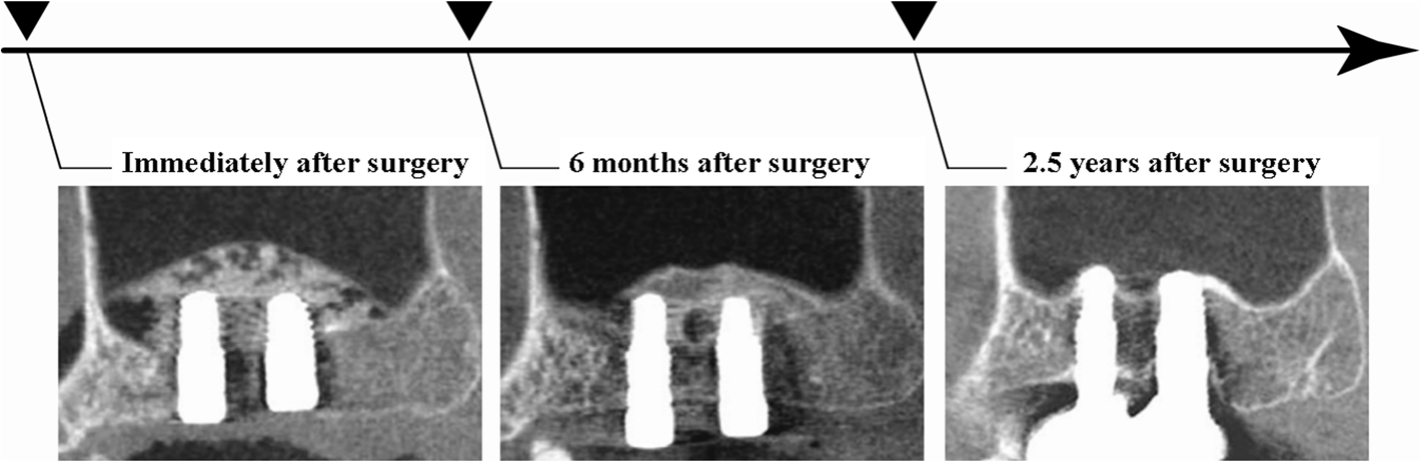
Treatment protocol for the present study. Postoperative CBCT was performed a minimum of three times, i.e., immediately, 6 months, and 2.5 years after implant placement

Patients underwent CBCT evaluations a total of four times: prior to surgery, immediately after surgery, 6 months after surgery, and 2.5 years after surgery. We used a dental CBCT device (Fine Cube; Yoshida, Japan). Imaging mode was set to the standard mode with voxel size at 0.146 mm^3^ and FOV of φ82.00 mm × 75.1 mm (Fig. 1).

In the present study, we used the superior spatial resolution of CBCT to measure changes over time in (a) the volume of the bone graft (BV) and (b) the height of the bone surrounding the implant (BH).

**Fig. 2:**
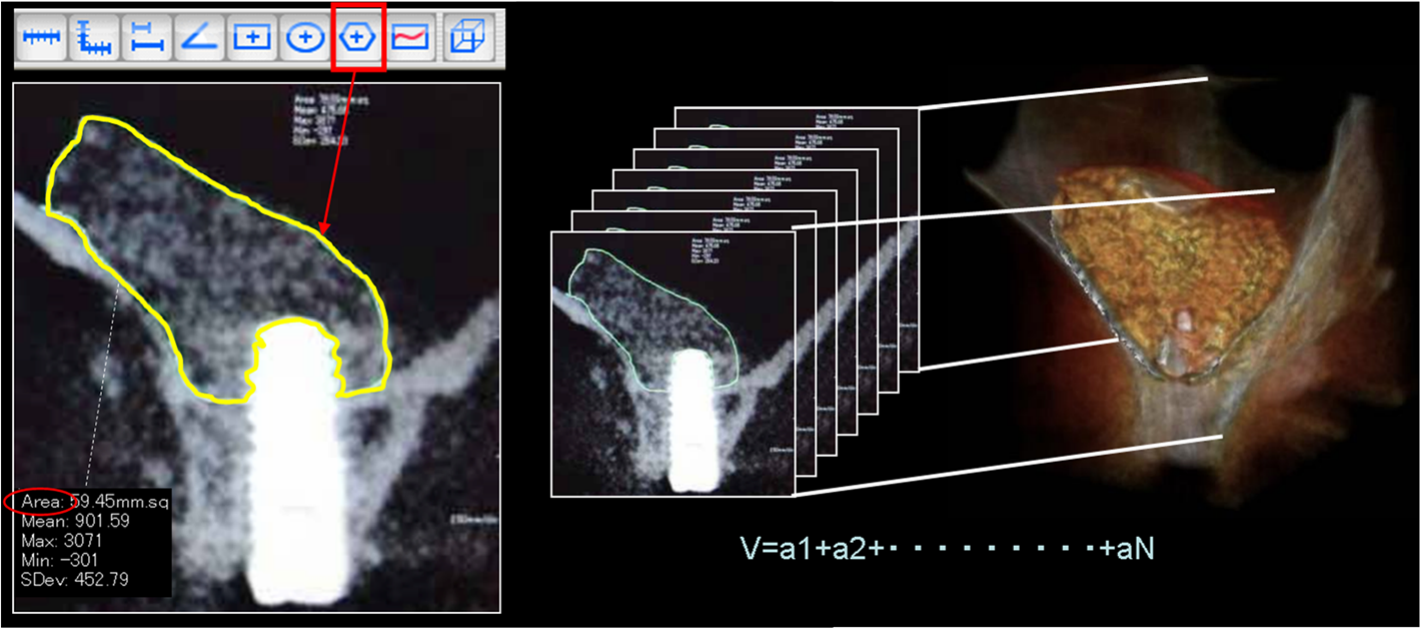
Radiographic examination of the volume of the bone graft (BV): Calculation of area on the frontal plane prior to and immediately after surgery using polygon tool. The polygon tool is included in the CT device, which was dragged around the perimeter of the target site to measure area. Graft volume calculation method (sum of the area and calculation of volume). Volume cm^3^ = area cm^2^ × *n* (number of images)
  The slice thickness (voxel value) was resized (0.146 mm → 1 mm) to derive the volume of the grafting agent on the basis of CBCT data. After setting the slice interval to 1 mm and specifying the output range (FOV option), the DICOM server for storing data was selected and registered.
  Next, image data of four time axes from prior to surgery, immediately after surgery, 6 months after surgery, and 2.5 years after surgery was simultaneously imported into a multi-data image analysis system. For confirming that the four images had the same positional relationship, we implemented synchronized scrolling after identifying similar anatomical reference points. The area of the implant site was measured using the polygon tool which was dragged around the perimeter of the target site to measure area. It allows measurements up to two places after the decimal point. It is operated by dragging the cursor only around the bone graft material site and avoiding the implant itself. In cases in which it was impossible to determine the margin between the implanted bone and the existing bone after 1-year examination, the entire bone was measured and the preoperative measurement was subtracted from the postoperative measurement.
  Then the images were taken at 1-mm increments mesio-distally, and the volume was calculated by adding the entire area of the bone grafting material. BoneVolume (BV) cm^3^ = area cm^2^ × *N* (number of images)
- The method of measuring the height of the bone surrounding the implant (BH) is described below (Fig. 3).

- The method of calculating the volume of the implant site is shown below (Fig. 2).

**Fig. 3:**
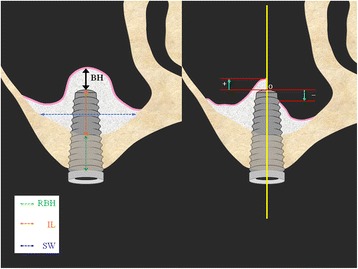
Radiographic examination of the height of the bone surrounding the implant (BH): Measurement of changes in the height of the implant tip to the bone fixation part over time in the frontal plane: the distance measured from the intersecting point of the long axis of the implant and the maxillary sinus floor to the implant tip: +maxillary side, −alveolar crest side. The liner valuables: residual bone height (RBH), implant length (IL), and width of sinus (SW)
  The measurements were taken at the long axis of the implant and on the frontal plane of the perpendicular frontal plane. In the event that there was grafted bone above the implant, the distance was measured from the point of intersection of the line connecting the buccal and palatal side of the maxillary sinus floor and the medial axis of the implant to the implant tip (+mm). Moreover, in the event that absorption of the grafting material exceeded the implant tip, the distance was measured from the point of intersection of the medial axis of the implant and the line connecting the most apical part of the grafted bone-to-implant contact (BIC) to the implant tip (−mm). In addition, the liner valuables (RBH, SW, IL) that may effect the outcomes of BH were measured. The following are the explanations of the linear valuables.
  Residual bone height (RBH): existing bone height to the maxillary sinus at the implant site
  Implant length (IL): the length of the part of the implant that projects into the maxillary sinus
  Width of sinus (SW): the width of the maxillary sinus from lateral wall to medial wall at the height of the center of the IL

### Statistical analysis

In the present study, the radiographic examinations were statistically analyzed for (a) volumetric changes of the bone graft (BV) and (b) changes in the height surrounding the implant (BH) over time. Moreover, it was examined whether the liner parameters (RBH, SW, IL), BH immediately after surgery (iBH), and the implant placement site affected the changes in BH over time.

Because the time CBCT was performed 2.5 years the surgery varied, analysis was performed using the generalized estimating equation (GEE). Thus, the time of CBCT was used as a variable to resolve the problem.

## Results

### Participants

The mean age of the 30 patients was 57.4 (40–75) years, and the male/female ratio was 4:26. The mean postoperative observation period was 3 years and 8 months (the maximum period was 5 years and 0 months; the minimum period was 2 years and 2 months). Total of 58 implants were placed at premolar and molar region. There were no dropouts, such as participants not visiting the clinic, during the observation period (Tables 1, 2, and 3).

**Table 1:** Age groups of the 30 patients

Age group (years)	Number of patients
40–49	7
50–59	10
60–69	10
70–79	3
Mean age 57.4 years	Total: 30 patients

**Table 2:** Observation period

Observation period (months)	Number of patients
12–18	11
19–24	4
25–30	3
31–36	1
37–42	3
43–48	2
49–54	2
55–60	4
Mean 3 years and 8 months	Total: 30 patients

**Table 3:** The number of implants according to site

Implant site	Number of implants
4	6
5	16
6	24
7	12
	Total: 58 implants

### Clinical evaluation

**Fig. 4:**
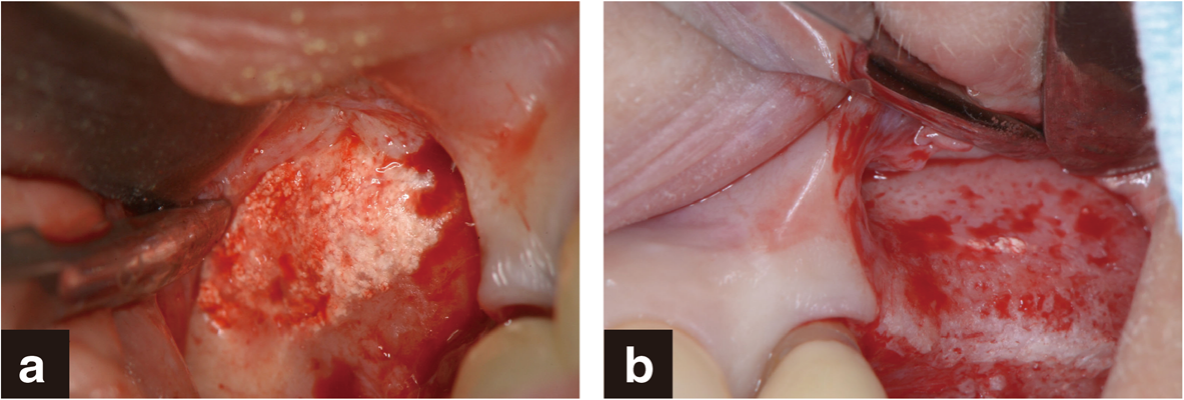
Clinical findings of the second surgery on biopsy at 6 months. The degree of residual grafting materials varied depending on the patient. **a** most of the β-TCP remained. **b** Replacement of the β-TCP by new bone had progressed

Implant osseointegration was achieved in all patients without any complications. During the observation period between January 2009 and January 2014, the implant survival rate was 100 %. At the time of second-stage surgery, macroscopic observation of the lateral wall of the maxillary sinus revealed differing degrees of β-TCP replaced by new bone (Fig. 4).

**Fig. 5:**
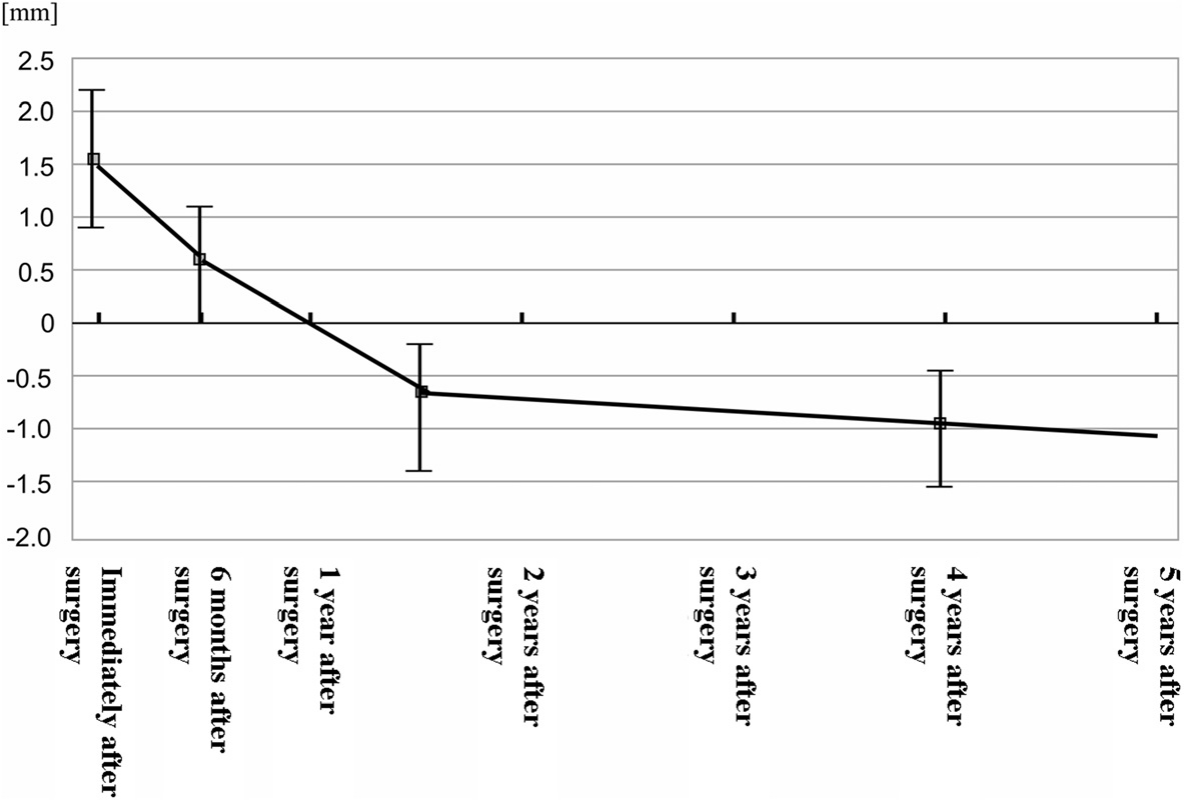
Radiographic examination (long-term changes in bone height surrounding the implant) *n* = 20 Number of implants. A total of 5 CBCT scans were taken prior to surgery, immediately after surgery, 6 months after surgery, 1–2 years after surgery, and 3–5 years after surgery

### Radiographic evaluation

In consideration of the radiation the patient would be exposed to and to increase the benefits to the patients, the fourth CBCT (2.5 years after surgery) was performed at a time when it did not overlap with radiation exposure that was conducted as part of complete physical examinations or required tests (periodontal disease of the remaining tooth, endodontic examinations, root fracture, etc.). Therefore, the fourth CBCT was performed at varying times. The distribution of observation time is shown in Table 4.

**Table 4:** The distribution of CBCT examination after 2.5 years

CBCT examination (year)	Number of patients
0–1	0
1–2	14
2–3	5
3–4	5
4–5	6
Mean 2.5	Total: 30

β-TCP could be differentiated from existing bone on CBCT even 6 months after surgery; however, the distinction became unclear 2.5 years after surgery, indicating that the replacement of the grafted bone by new bone appeared to have been accelerated. The effect of implant artifacts made it difficult to measure changes in the bone density index (gray scale value).

- Volumetric changes in the graft: The mean β-TCP volume decreased from immediately after surgery (1206.9 ± 437 cm^3^) to 6 months after surgery (912.6 ± 356 cm^3^). It further decreased 2.5 years after surgery (662 ± 294 cm^3^), and the reduction in volume was observed in all patients. Percent volume changes 6 months after surgery and 2.5 years after surgery using volume immediately after surgery as the baseline value were 75.6 and 54.9 %, respectively (Table 5).

**Table 5:** Radiographic examination of BV (volumetric changes in graft bone over time)

BV	Mean	Reduction rate
Immediately after surgery (*n* = 30)	1206 ± 437 (477 to 2292)	100 %
6 months after surgery (*n* = 30)	912 ± 356 (428 to 1760)	75.6 %
2.5 years after surgery (*n* = 30)	662 ± 294 (204 to 1568)	54.9 %

*BV* bone volume, *n* = 30: the number of maxillary sinuses

Reduction rate: provided that volume was 100 % immediately after surgery

**Fig. 6:**
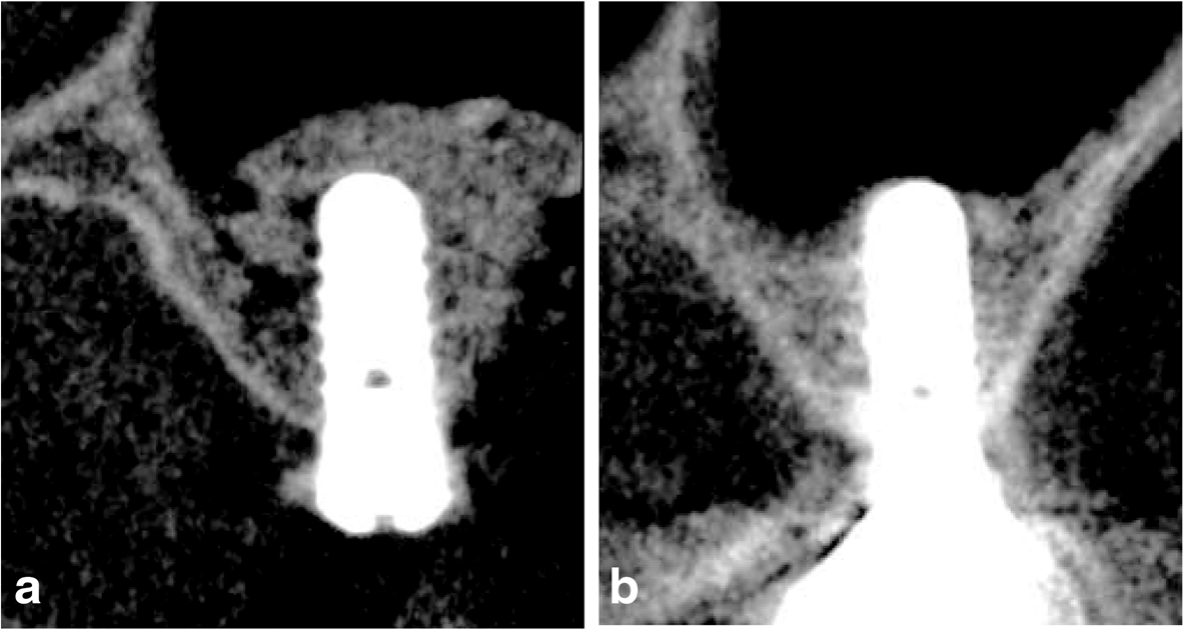
Radiographic examination: The relationship between changes in the maxillary sinus floor associated with a reduction in the grafted bone and the implant tip (**a** immediately after surgery, **b** 5 years after surgery)

Analysis using generalized estimating equations (GEE) with patient clusters
- Change in height of bone surrounding the implant (height from the maxillary sinus floor to the implant tip): Immediately after surgery, all patients had grafted bone between the implant tip and the maxillary sinus floor, with a mean of 2.00 ± 1.51 mm. This height decreased to 0.73 ± 1.33 mm at 6 months after surgery and −0.72 ± 1.11 mm at 2.5 years after surgery (Fig. 5). 41/58 implants had developed pneumatization 2.5 years after surgery and had no bone around the implant tip. The overall mean reduction in bone height was 2.72 ± 1.27 mm: (i) the mean reduction from immediately after surgery to 6 months afterward was 1.28 ± 1.05 mm and (ii) that from 6 months after surgery to 2.5 years after surgery was 1.44 ± 1.45 mm (Table 6, Fig. 6).

**Table 6:**
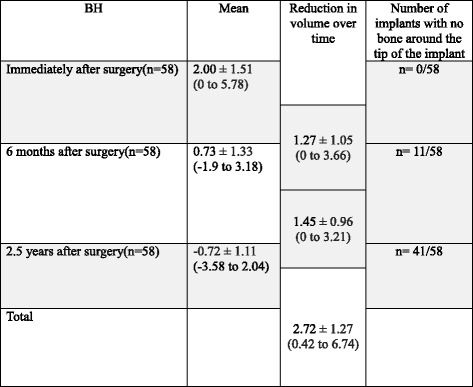
Radiographic examination of BH (changes in bone height surrounding the implant)

*BH* bone height surrounding the implant, *n* = 58: number of implants

Analysis using generalized estimating equations (GEE) with patient clusters

The liner parameters: mean RBH, IL, and SW were 4.48 ± 1.51 mm, 6.50 ± 1.88 mm, and 10.32 ± 2.29 mm with a mean value, respectively (Table 7). In addition, on examining the 10 patients (20 implants) with multiple long-term data of ≥1 year after surgery, the height reduction was found to have progressed to the same extent from approximately 1–2 years after surgery, after which there was only a slight change (Fig. 5).

**Table 7:** The radiographic measurements of liner parameters at immediately after surgery (RBH, IL, SW)

Parameter	Mean (mm)	SD (mm)
RBH	4.48	1.51
IL	6.50	1.88
SW	10.32	2.29

### Statistical analysis

Radiographic evaluation items were statistically analyzed: (a) a significant decrease in graft volume (BV) was observed over time (*p* < 0.00) and there was no statistically significant effect of age or gender and (b) a significant decrease in bone height (BH) was observed over time (*p* < 0.001) and there was no statistically significant effect of age or gender. We found that this change of BH was affected by RBH (*p* = 0.003) and IL (*p* = 0.001); the thicker the RBH, the less the decrease of BH; the longer the IL, the more the decrease of BH over time. We determined that the higher the immediately postoperative bone height (iBH) was, the higher the subsequent total height would be. We also observed that the coefficient for the interactive items tended to have negative values and that the higher the postoperative bone height was, the larger the amount of decrease was. There was no significant difference in the change in bone height according to SW and also no significant difference according to implant site (Wald chi-square value = 1.221, *P* = 0.748) (Table 8).

**Table 8:** Examination of the impact of RBH, IL, SW, and iBH in the height from the implant tip to the bone integration site (BH)

	Coefficient	Standard error	95 % CI	*P* value
Time (months)	−0.087	0.012	−0.112, −0.063	<0.001
RBH	−0.016	0.118	−0.247, 0.215	0.891
Interaction terms (RBH × time)	0.007	0.002	0.002, 0.011	0.003*
Time (months)	−0.016	0.011	−0.039, −0.006	0.157
IL	0.013	0.104	−0.190, 0.216	0.898
Interaction terms (IL × time)	−0.007	0.002	−0.010, −0.003	0.001*
Time (months)	−0.043	0.021	−0.085, −0.002	0.041
SW	−0.076	0.073	−0.220, 0.067	0.295
Interaction terms (SW × time)	−0.001	0.002	−0.005, 0.003	0.549
Time (months)	−0.026	0.006	−0.038, −0.013	<0.001
iBH	0.625	1.293	0.372, 0.879	<0.001
Interaction terms (iBH × time)	−0.005	0.003	−0.012, 0.001	0.080

*RBH* residual bone height, *IL* implant length, *SW* width of sinus, *iBH* the height of bone around the tip of implant immediately after surgery. Analysis using generalized estimating equations (GEE: *p < 0.05)

## Discussion

As grafting materials for maxillary sinus floor augmentation, autogenous bone, which is considered as the gold standard and reported first by Boyne et al. using iliac bone graft, has been used as the first-choice material. In terms of osteogenic, osteoinductive, and osteoconductive properties, autogenous bone is considered ideal; however, the use of autogenous bone places great physical stress on patients because of the need for surgery at the donor site. Synthetic bone substitutes such as β-TCP are clinically very beneficial because donor site surgery can be avoided. Moreover, in the 1996 Sinus Consensus Conference, these materials were concluded as having highly predictable outcomes. Although there are many related factors, no difference in the success rate has been observed according to the grafting material [14].

Artzi et al. conducted a comparative experiment of bovine bones and β-TCP to fill defect areas in dogs. They reported that β-TCP was absorbed within 24 h and replaced by autogenous bone, whereas with bovine bone, approximately 30 % granules remained [15]. Thus, β-TCP is considered the ideal grafting material.

The permanence of the new bone formed within the maxillary sinus has been primarily evaluated in humans using radiography. The clinical assessment and short-term and long-term investigations have also been conducted using panoramic radiography and conventional CT [8–12]. Panoramic radiography analysis does not have the shortcomings of radiation risk and expensive CT equipment; therefore, images can generally be obtained frequently. However, its limitations include the ability to assess two-dimensional height, and various magnifications or errors, such as distortion, must also be taken into consideration. In the past, evaluations during implant treatment were primarily performed using panoramic radiography and conventional CT. Recently, however, the use of preoperative and postoperative CBCT has gained popularity, and radiographic assessment by CBCT for volumetric changes in graft bone in maxillary sinus floor augmentation has also been reported [16]. According to our radiographic analysis by CBCT of dental implantation using β-TCP in the present study, we found that the graft volume decreased over time, both at 6 months after surgery and even at 2.5 years after surgery. The result of 54.9 % graft volume change of 2.5 years after surgery is similar to previous reports [17]. Loads were applied to the implant only 6 months after surgery. Nevertheless, the results suggested that it is difficult to control maxillary sinus pneumatization by exerting implant occlusive load. Zijderveld et al. conducted a 5-year radiographic study using panoramic radiography to evaluate periodic changes in β-TCP [20 patients: 10 patients with autogenous bone (taken from the mental region) and 10 patients with β-TCP]. They reported that the majority of β-TCP absorption occurred within the first 7.5 months and that very little change was observed after 1.5 years [12]. Furthermore, in 2004, Hatano et al. used a 2:1 mixture of autogenous bone-to-bovine bone and reported that the maxillary sinus floor was situated at the same height or lower than the implant tip in most patients [11]. These results are similar to our study that maxillary sinus pneumatization continues to progress ≥1 year after surgery; it stabilizes 3 years after surgery and the implant tip protruded beyond the maxillary sinus floor in approximately 70 % implants (41/58 implants) in patients who were followed up 2.5 years after surgery. However, there were no clinical abnormalities, such as maxillary sinusitis. With regard to liner valuables (RBH, IL, SW, iBH) in the present study, the results suggested that it is difficult to control maxillary sinus pneumatization. There was no significant difference in the change in bone height according to SW, and this result is inconsistent with previous reports [18]. There was a limited number of subjects in this study; we believe it is necessary to conduct long-term tests of larger numbers of subjects in the future.

During the observation period, there was no clinical implant failure and the suvival rate for implantation and maxillary sinus floor augmentation was 100 %. Histological examination in a previous report on maxillary sinus augmentation using β-TCP (Cerasorb®), Szabo et al. compared bilateral maxillary sinus augmentation in the same patients using β-TCP and iliac bone at 6 months after surgery and reported a new bone ratio of 36.5 % in the β-TCP group (control group: 38.3 % autogenous bone), and in a similar experiment, Suba et al. reported a new bone ratio of 32.4 % in the β-TCP group (control group: 34.7 % autogenous bone), which were similar outcomes to those for the autogenous bone. These histological examinations provided supporting evidence that during the year after grafting, β-TCP is gradually replaced by new bone over time [19–21]. Our clinical and radiographic examination of maxillary sinus floor augmentation using β-TCP suggests that β-TCP is a very useful synthetic bone substitute with very similar progress to autogenous graft. It is of our interest to further continue our long-term clinical study.

## Conclusions

- Maxillary sinus graft augmentation using β-TCP is clinically effective.
- Analysis by CBCT provides superior spatial resolution and allows for extremely accurate postoperative evaluation of maxillary sinus floor augmentation and bone volume measurements.
- On the basis of CBCT examinations, although maxillary sinus pneumatization continues to progress ≥1 year after surgery, it stabilizes 3 years after surgery.
